# Numerical Investigation of Heat Transfer Enhancement in a Rectangular Heated Pipe for Turbulent Nanofluid

**DOI:** 10.1155/2014/369593

**Published:** 2014-08-26

**Authors:** Hooman Yarmand, Samira Gharehkhani, Salim Newaz Kazi, Emad Sadeghinezhad, Mohammad Reza Safaei

**Affiliations:** ^1^Department of Mechanical Engineering, University of Malaya, 50603 Kuala Lumpur, Malaysia; ^2^Young Researchers and Elite Club, Islamic Azad University, Mashhad Branch, Mashhad, Iran

## Abstract

Thermal characteristics of turbulent nanofluid flow in a rectangular pipe have been investigated numerically. The continuity, momentum, and energy equations were solved by means of a finite volume method (FVM). The symmetrical rectangular channel is heated at the top and bottom at a constant heat flux while the sides walls are insulated. Four different types of nanoparticles Al_2_O_3_, ZnO, CuO, and SiO_2_ at different volume fractions of nanofluids in the range of 1% to 5% are considered in the present investigation. In this paper, effect of different Reynolds numbers in the range of 5000 < Re < 25000 on heat transfer characteristics of nanofluids flowing through the channel is investigated. The numerical results indicate that SiO_2_-water has the highest Nusselt number compared to other nanofluids while it has the lowest heat transfer coefficient due to low thermal conductivity. The Nusselt number increases with the increase of the Reynolds number and the volume fraction of nanoparticles. The results of simulation show a good agreement with the existing experimental correlations.

## 1. Introduction

Since nanofluids have shown the capability of transmitting heat more than the conventional fluids, researchers are interested in thermal conductivity which could be useful in many applications including air conditioning and refrigeration. Thus much research has been focusing on this area. Kulkarni et al. [1] represented the use of nanofluids to retard the decrease of cogeneration efficiency. The thermal conductivity of Cu-water nanofluids produced by chemical reduction process was assessed by Liu et al. [2]. The investigation results show 23.8% enhancement of thermal conductivity at 0.1% volume fraction of copper particles. Furthermore, it was represented that thermal conductivity increases with particle volume fraction. Hwang et al. [3] reported that the thermal conductivity improvement of nanofluids was remarkably affected by thermal conductivity of both base fluids and nanoparticles. As an example, thermal conductivities of nanofluids produced by multiwalled carbon nanotube and water are greater than those of SiO_2_-water nanofluids [4]. Yoo et al. [5] pointed out that surface to volume ratio of nanoparticles could be a main factor of influencing the nanofluids thermal conductivity. The thermal conductivity of Cu-water nanofluid was measured by Jana et al. [6]. On the other hand, Kang et al. [7] found a 75% thermal conductivity improvement for ethylene glycol with 1.2% diamond nanoparticles of 30 to 50 nm diameter. In contrast with these improvements, there are some researchers who found some abnormal results. Trivedi [8] found that decreasing the temperature of a component increases its performance such as reliability. Nanofluids have shown their capability as a new generation of coolants for many technological services such as automobile thermal management due to their greater thermal conductivities than the base fluids [9–11]. Vajjha and Das [4] presented dependency of thermal conductivity on both temperature and nanoparticles concentration. They found that it would be more beneficial if nanofluids are employed at high temperature applications. Peng et al. [12] observed that the frictional pressure drop of nanorefrigerant boiling flow inside a horizontal smooth pipe is more than that of conventional pure refrigerant which subsequently increases with the increase of volume fraction of nanoparticles. Xie et al. [13] noticed the effect of particle shape on the thermal conductivity. The results were compared with those of suspensions of different geometric shape of the particles with the similar material and base fluid. The report illustrated that the lengthened particles show better enhancement of the thermal conductivity. Touloukian and Makita [14] found experimentally that the volume fraction increase or the nanoparticles size decrease enhances the raise of nanofluid average temperature and decreases the heat transfer coefficient ratio of nanofluids and base fluids. Khanafer et al. [15] studied the heat transfer augmentation in a two-dimensional enclosure by using Nanofluid. The viscosity of the nanofluids was measured by Pantzali et al. [16] and showed double fold increase in comparison with pure water. An increase in the pressure drop and therefore increase in the pumping power were observed. They obtained that the pumping power was increased about 40% compared to water for a given flow rate.

Haghshenas Fard et al. [17] studied heat transfer efficiency numerically in case of laminar convective heat transfer to nanofluids. They found that the heat transfer coefficient of nanofluids increases with the rise of volume fraction of nanofluids and Peclet number. Similarly laminar mixed convection of Al_2_O_3_-water nanofluid in a horizontal tube under heating at the top half surface of a copper tube was investigated numerically by Allahyari et al. [18]. They observed that increasing of the nanoparticle concentration had remarkably enhanced the heat transfer coefficient whereas the skin friction coefficient was not considerably influenced. The natural convection in an isosceles triangular enclosure was simulated by Aminossadati and Ghasemi [19]. A heat transfer enhancement was observed by them when the solid volume fraction and Rayleigh number were increased. Mahmoudi et al. [20] simulated a cooling system which had been working with natural convection, and they have concluded with a statement that the average Nusselt number increases linearly with the increase of solid volume fraction of nanoparticles. Mansour et al. [21] numerically studied a mixed convection flow in a square lid-driven cavity partially heated from below and filled with different nanofluids to observe the effect of particles type and concentration on heat transfer. They reported that increase in solid volume fraction raises the corresponding average Nusselt number. Shahi et al. [22] analyzed the heat transfer enhancement of a nanofluid by simulation of an annular tube driven by inner heat generating solid cylinder. It has been shown that the average Nusselt numbers were increasingly depending on the solid concentration in the suspension. In addition, Izadi et al. [23] worked on forced convection Al_2_O_3_-water nanofluid flow in an annular tube by simulation. In general the higher nanoparticle volume fraction is added to base fluid, and the more convective heat transfer coefficient is resulted.

Namburu et al. [24] simulated turbulent flow and heat transfer enhancement for three kinds of nanoparticles added to both ethylene glycol and water mixture flowing through a circular pipe. In this study *κ*-*ε* turbulent model proposed by Launder and Spalding [25] was adopted. The conclusions illustrated that an increase in concentration of nanofluid is led to rise of the average Nusselt number. Lotfi et al. [26] reported the effect of different models of nanoparticle simulation on forced convection turbulent flow in a circular tube. They made comparisons among three different single-phase, two-phase mixture and Eulerian models. Comparison of the experimental values showed that the mixture model is the most accurate one. Ghaffari et al. [27] studied numerically the turbulent mixed convection heat transfer to Al_2_O_3_-water nanofluid flowing through a horizontal curved pipe with particles size of about 28 nm. The effect of the buoyancy force, centrifugal force, and nanoparticles concentration are assessed in this study. The result illustrated that increases of the nanoparticle volume fraction enhanced the Nusselt number even though its impact on the skin friction coefficient was not remarkable. The turbulent flow of nanofluids with different volume fractions of nanoparticles flowing through a two-dimensional duct under constant heat flux condition was simulated by Rostamani et al. [28]. The results show that both the Nusselt number and the heat transfer coefficient of the nanofluid are strongly dependent on nanoparticles and increased by increasing of the volume concentration of nanoparticles in the suspension. In addition the results presented that, by increasing the volume fraction, the shear stress increases. Recently Fotukian and Nasr Esfahany [29] investigated the turbulent convective heat transfer of dilute *γ*-Al_2_O_3_/water nanofluid inside a circular tube experimentally and found that increasing the volume fraction of nanoparticles in the range of less than 0.2% provides no significant influence on heat transfer enhancement.

In this paper, an effective single-phase model was applied to study the turbulent forced convection flow of a nanofluid in a uniformly heated rectangular tube. The influence of particles volume fraction and Reynolds number is also studied for each type of particles.

## 2. Methodology

### 2.1. Governing Equations

It is very important to set the governing equations (momentum, continuity, and energy) to complete the CFD analysis of the rectangular channel. The phenomenon under consideration is governed by the steady two-dimensional form of the continuity, the time-averaged incompressible Navier-Stokes equation, and energy equation. In the certain tensor systems these equations can be written as

 continuity equation:
  (1) $$\begin{array}{c} \nabla \cdot \left(\rho_{\text{eff}}U\right)=0, \end{array}$$
 momentum equation:
  (2) $$\begin{array}{c} \nabla \cdot \left(\rho_{\text{eff}}UU\right)=-\nabla P+\nabla \left(\tau +\tau^{T}\right), \end{array}$$
 energy equation:
  (3) $$\begin{array}{c} \nabla \cdot \left(\rho_{\text{eff}}c_{p,\text{eff}}\overset{-}{T}\;\overset{-}{U}\right)=\nabla \cdot \left(k_{\text{eff}}\left(\nabla \overset{-}{T}\right)\right)-\left(\overset{-}{U^{\prime }T^{\prime }}\right). \end{array}$$

The standard *k*-*ε* model was employed according to Launder and Spalding [25]:

(4) ∇·ρeffkU∇·μeff+μtσk∇k+Gk−ρeffε,∇·ρeffεV∇·μeff+μtσε∇ε+C1εεkGk−C2ερeffε2k,Gk−ρeffui′uj′−∇U,μtρeffCμk2ε,

where *C*_*μ*_ = 0.09, *σ*_*k*_ = 1.00, *σ*_*ε*_ = 1.30, *C*_1*ε*_ = 1.44, and *C*_2*ε*_ = 1.92.

### 2.2. Physical Properties of Nanofluid

In order to conduct numerical simulation for nanofluids, the effective thermophysical properties of nanofluids must be determined first. In this study, the nanoparticles being used are Al_2_O_3_, ZnO, CuO, and SiO_2_. Thermophysical properties of base fluid and the nanoparticles which are used in the present work are reported in Table 1.

Generally the required properties for numerical simulations are effective thermal conductivity (k_eff_), effective dynamic viscosity, effective mass density (*ρ*_eff_), and effective specific heat (*C*_*p* eff_). All these effective properties are truly calculated based on the mixing theory.

The density of nanofluid, *ρ*_nf_, can be obtained from [32]

(5) $$\begin{array}{c} \rho_{\text{nf}}=\left(1-\phi \right)\rho_{f}+\phi \rho_{s}, \end{array}$$

where *ρ*_*f*_ and *ρ*_np_ are the mass densities of the based fluid and the solid nanoparticles, respectively.

The effective heat capacity at constant pressure of nanofluid, (*ρC*_*p*_)_nf_, can be calculated from [33]

(6) $$\begin{array}{c} {\left(\rho C_{p}\right)}_{\text{nf}}=\left(1-\phi \right){\left(\rho C_{p}\right)}_{f}+\phi {\left(\rho C_{p}\right)}_{s}, \end{array}$$

where (*ρC*_*p*_)_*f*_ and (*ρC*_*p*_)_*s*_ are heat capacities of the based fluid and the solid nanoparticles, respectively.

The effective viscosity, (7), can be obtained by using the mean empirical correlations suggested by Bianco et al. [33]:

(7) μeff=μf∗11−34.87dp/df−0.3∗∅1.03df=6MNπρfo,

where *M* is the molecular weight of the base fluid, *N* is the Avogadro number = 6.022 × 10^23^ moL^−1^, and *ρ*_fo_ is the mass density of the base fluid calculated at temperature *T*_0_ = 300 K. It could be noticed that, for simplicity in this study, the changes of viscosity with temperature along the tube are neglected and the viscosity at all points is considered to be the same as the viscosity at inlet temperature.

By considering Brownian motion of nanoparticles in channel, the effective thermal conductivity can be obtained by using the mean empirical equations (8), (9), and (10) [4]:

(8) $$\begin{array}{c} K_{\text{eff}}=k_{\text{static}}+k_{\text{Brownian}}, \end{array}$$

where *k*_static_ indicates the thermal conductivity improvement from the advanced thermal conductivity of nanoparticles and *k*_Brownian_ represents the effect of Brownian motion of particles. *k*_Brownian_ has also considered the influence of movement of fluid particles with nanoparticles. Consider the following:

(9) $$\begin{array}{c} k_{\text{static}}=k_{f}\left[\frac{\left(k_{s}+2k_{f}\right)-2\emptyset \left(k_{f}-k_{s}\right)}{\left(k_{s}+2k_{f}\right)+\emptyset \left(k_{f}+k_{s}\right)}\right], \end{array}$$

where *k*_*s*_ and *k*_*f*_ are thermal conductivities of the nanoparticles and the base fluid, respectively. Consider the following:

(10) $$\begin{array}{c} k_{\text{Brawnian}}=5\times 10^{4}\beta \emptyset \rho_{f}C_{p,f}\sqrt{\frac{K_{B}T}{2\rho_{s}\cdot R_{s}}}f\left(T,\emptyset \right), \end{array}$$

where *ρ*_*f*_ and *ρ*_*s*_ are the densities of the base fluid and the particles, respectively, and *C*_*p*,*f*_ is the specific heat capacity of the base fluid. *K*_*B*_ is the Boltzmann constant, 1.381 × 10^−23^ J/k. *β* is a parameter which indicates the effect of interaction between nanoparticles and the movement of fluid around the particles. *f*(*T*, *∅*) represents the temperature dependency of nanofluids, where both *f*(*T*, *ϕ*) and *β* were obtained by utilizing the existing experimental data.

The modified *f*(*T*, *ϕ*) equation was reported by Vajjha and Das [4]:

(11) fT,ϕ=2.8217×10−2ϕ+3.917×10−3T273.15+−3.0669×10−2ϕ−3.91123×10−3.

### 2.3. Simulation Cases

A rectangular pipe with constant heat flux at the wall is considered in this study. The effects of various types of nanofluids are investigated under different volume fractions (1–5%) and Reynolds number in the range of 5000 to 25000. Fluid at the entrance has been considered as a constant temperature of 300 K and uniform axial velocity.

### 2.4. Numerical Methods

In the present study of heat transfer to turbulent nanofluids in a rectangular duct, the standard *k*-*ε* turbulent model and the Renormalized Group *k*-*ε* turbulence method were used. The time independent incompressible Navier-Stokes equations and the turbulence model analysis were solved by using finite volume method. To evaluate the pressure field, the pressure-velocity coupling algorithm SIMPLE (semi-implicit method for pressure-linked equations) was chosen. The solutions are considered to be converged when the normalized residual values reach ≤10^−5^ for all parameters.

### 2.5. Geometry Structure and Boundary Conditions

According to the proposed geometry, the cross section and length are 0.01 m^2^ and 2 m respectively. The geometry is simplified to 2-dimenssional planner structure and only half of the pipe is considered for simulation as the upper and lower parts are symmetrical.

**Figure 1:**
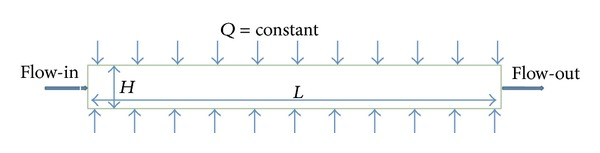
Schematic diagram of the rectangular channel.

**Figure 2:**
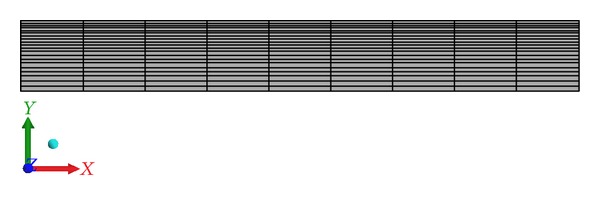
Mesh layout of present geometry, axisymmetric about *X*-axis.

The boundary conditions and grid layout for this study are specified for the computational domain as shown in Figures 1 and 2. The top wall is subjected to a constant heat flux of 20000 (W/m^2^) and the bottom wall is symmetry to the top wall. The left side is subjected to velocity inlet and the right side is subjected to pressure outlet.

## 3. Results and Discussion

### 3.1. Mesh Dependency Test and Validation

**Figure 3:**
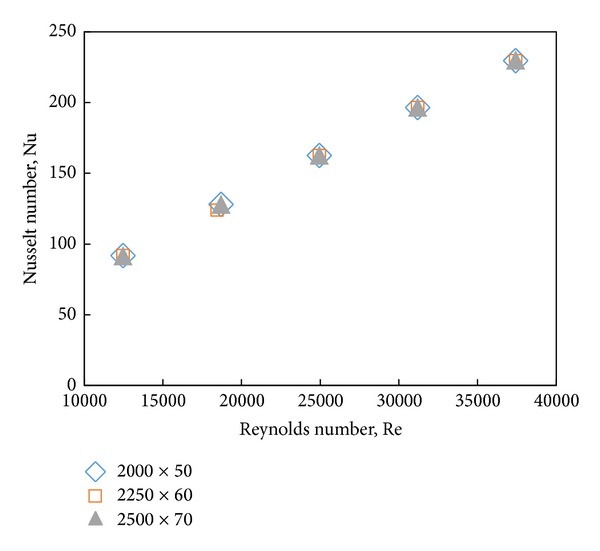
Comparison of the Nusselt number for water with different grids.

Several different grid distributions have been tested to ensure that the calculated results (Nu numbers) are grid independent (Figure 3). The Nu numbers are calculated based on the simulation results as follows:

(12) $$\begin{array}{c} \text{Nu}=\frac{h\cdot D_{h}}{k_{\text{eff}}}=\frac{q^{\prime \prime }\cdot D_{h}}{\left(T_{w}-T\right)\cdot k_{\text{eff}}}, \end{array}$$

where *h*, *D*_*h*_, *T*_*w*_, and *q*′′ are heat transfer coefficient, hydraulic diameter, wall temperature, and heat flux, respectively.

Equations (13)–(17) are used for evaluation of Nusselt number for water and nanofluids. Here (15)–(17) are specifically used for calculations of Nusselt number of water whereas (13)–(17) are applied for calculating the Nusselt number of nanofluids.

Maïga et al. [34] equation for nanofluid:

(13) Nu=0.085 Re0.71Pr0.35.

Pak and Cho [35] equation for nanofluid:

(14) Nu=0.021 Re0.8Pr0.5.

Dittus and Boelter [36] equation for water and nanofluid:

(15) Nu=0.023 Re0.8Pr0.4.

Bejan [37] equation for water and nanofluid:

(16) Nu=0.021Re0.87−280Pr0.4.

Gnielinski [38] equation for water and nanofluid:

(17) Nu=f/8Re−1000Pr1+12.7f/80.5Pr2/3−1,

where *f* = (0.79ln⁡⁡*Re*−1.64)^−2^.

According to Figure 3, it is observed that the calculated Nusselt number for base fluid (water) is independent of the number of grid points. The selected grid consists of 2000 and 50 nodes in the *x* and *y* directions, respectively. The maximum deviation of the selected mesh relative to the two other finer meshes (i.e., 22250 × 60 and 2500 × 70) is less than 0.6%.

**Figure 4:**
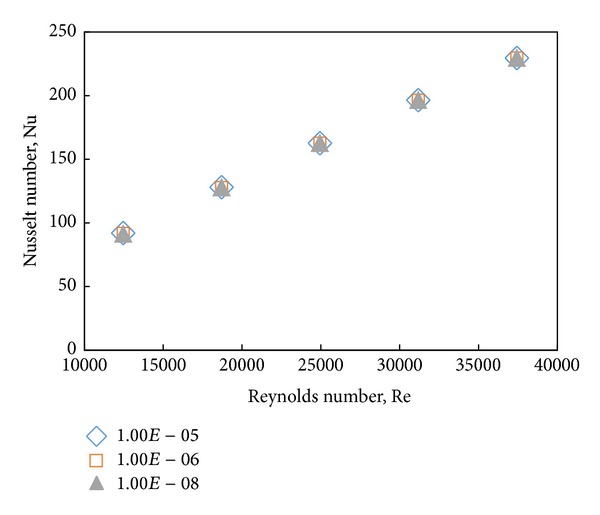
Residual sensitivity test for water.

The selected residual value is 10^−5^ for less computational effort. Since the less residual value might influence the accuracy of the results, the residual sensitivity test has been done to assess the effect of this value. According to Figure 4 the predicted Nu number of water by the CFD model considering residual value of 10^−5^ shows no significant deviation from those with 10^−6^ and 10^−8^ residual values.

**Figure 5:**
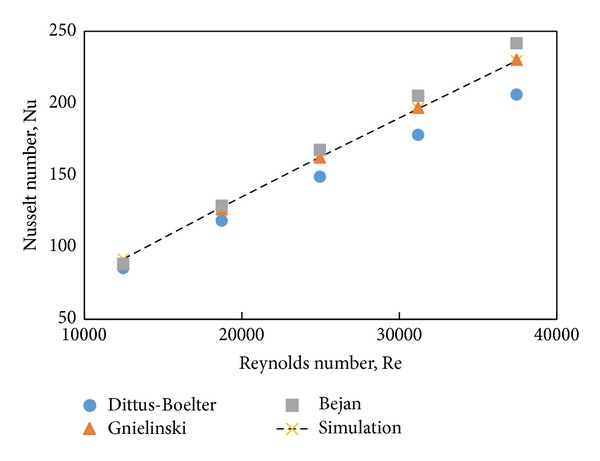
Comparison of Nusselt number from computed values and benchmarks for water.

To justify the computational model, evaluated numerical results are compared with the calculated data from some empirical correlations.  Figure 5 represents the comparison between the evaluated Nusselt number and the calculated values from the three different empirical correlations such as Gnielinski [38], Dittus and Boelter [36], and Bejan [37]. It can be seen that the numerical Nusselt numbers for water are in a good agreement with the benchmarks. There is a slight deviation which could be considered negligible in validation. The average deviation of estimated data and Dittus and Boelter data is 9.61% but the average deviation of Gnielinski and Bejan correlation data and simulation result is 0.45% and 5%, respectively, indicating that the model could be used for heat transfer calculations with reasonable accuracy.

### 3.2. Effect of Nanoparticles with Different Volume Concentrations on the Nusselt Number and Heat Transfer Coefficient

**Figure 6:**
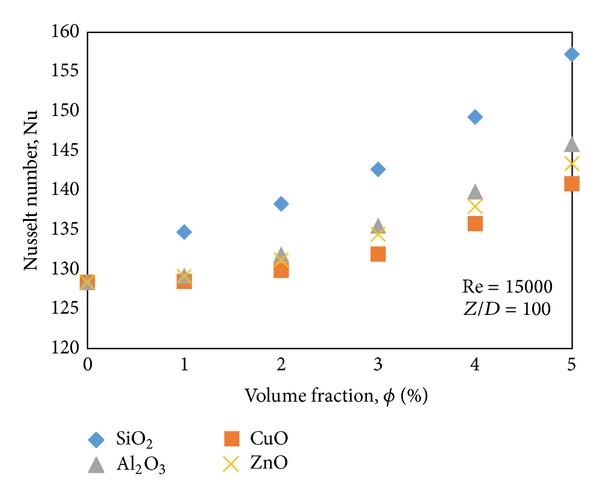
Effect of volume concentrations of different nanoparticles on Nusselt number.

The effect of volume concentrations of different types of nanoparticles on the Nusselt number is presented in Figure 6. The Reynolds number is 15000 and the volume concentrations varied as 1%, 2%, 3%, 4%, and 5%. The results show that with the increase of concentration of nanoparticles in the base fluid the Nusselt number increases. In addition SiO_2_-water nanofluid shows the most enhancement of Nusselt number, while the other nanoparticles (Al_2_O_3_, ZnO, and CuO) have little variation of Nusselt numbers at all the volume fractions. It should be noted that the Gnielinski formula is more accurate than Dittus and Boelter correlation, so the discrepancies between simulated results and Gnielinski equation are presented in Table 2. As it is illustrated simulated results are in good agreement with the correlations.

**Figure 7:**
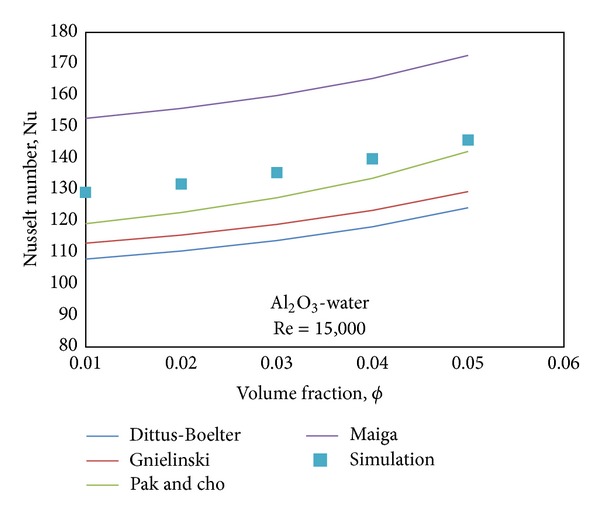
Comparison between the computed data of Nusselt numbers and the data from four benchmarks at different concentrations for Al_2_O_3_-water.

In addition to this, for more comprehensive comparison, the simulation results for Al_2_O_3_-water nanofluid at *Re* = 15,000 and at different nanoparticle concentrations (1%–5%) are benchmarked against the data from some empirical correlations (i.e., [34–36, 38]). According to Figure 7, although the simulation results give a good agreement with the all correlations, as expected, the Pak and Cho correlation shows the closest answer to the simulation results.

**Figure 8:**
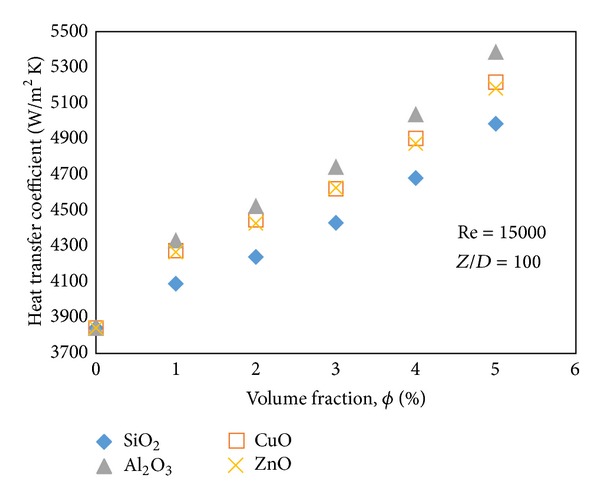
Effect of volume concentrations of nanoparticles on heat transfer coefficient.

Figure 8 shows the effect of different concentrations of the selected nanoparticles on heat transfer coefficient. Since the dimensionless function Nusselt number shows the ratio of convection mechanism to conduction one for each kind of fluid, it is not reliable for comparison of only Nusselt number of different nanofluids. For instance, SiO_2_ has the maximum Nusselt number while it shows the least heat transfer coefficient due to the lowest thermal conductivity. In this case Al_2_O_3_ shows the maximum heat transfer coefficient while CuO and ZnO are after it with a slight difference.

### 3.3. The Effect of Nanoparticles at Different Reynolds Numbers on the Nusselt Number and Heat Transfer Coefficient

The effective thermophysical properties of the different nanofluids are used at a constant volume fraction of 3% but at different inlet velocities or Reynolds numbers varied from 5000 to 25000.

**Figure 9:**
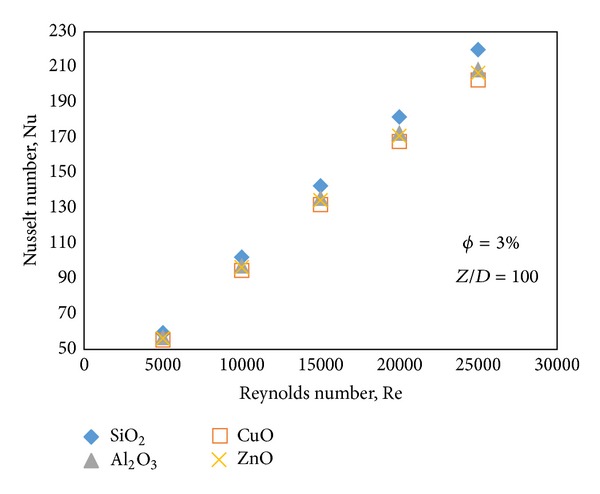
Nusselt number of different types of water based nanofluids at different Reynolds numbers.

**Figure 10:**
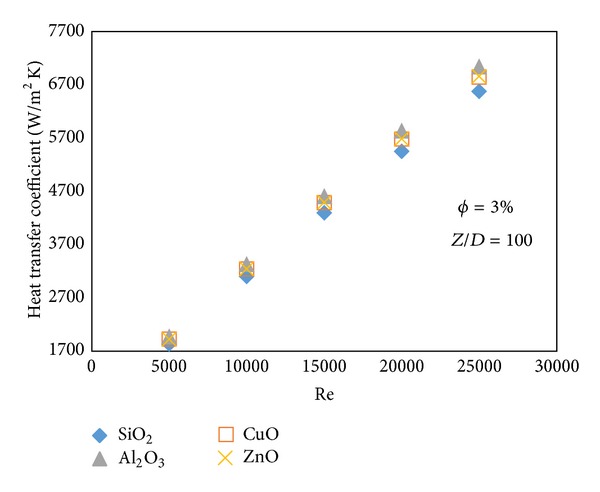
Heat transfer coefficient of different types of water based nanofluids at different Reynolds numbers.

Figures 9 and 10 illustrate that with the increase of Reynolds number the Nusselt number and heat transfer coefficient also increase. Similar to the results obtained in the Figures 6 and 8, where SiO_2_-water has the highest Nusselt number but it shows the least heat transfer coefficient. Slopes of the graphs in Figures 9 and 10 are higher than slopes of the graphs in Figures 6 and 8, so it can be concluded that the changes of Nusselt number and heat transfer coefficient are more sensitive to the Reynolds number than the volume fraction.

Moreover, discrepancies between simulated results and Gnielinski correlation for SiO_2_-water are shown in Table 3. According to the obtained discrepancies, it is concluded that the use of Gnielinski correlation to calculate the Nusselt number for nanofluids is more practical for Reynolds number greater than 10^4^ [24].

**Figure 11:**
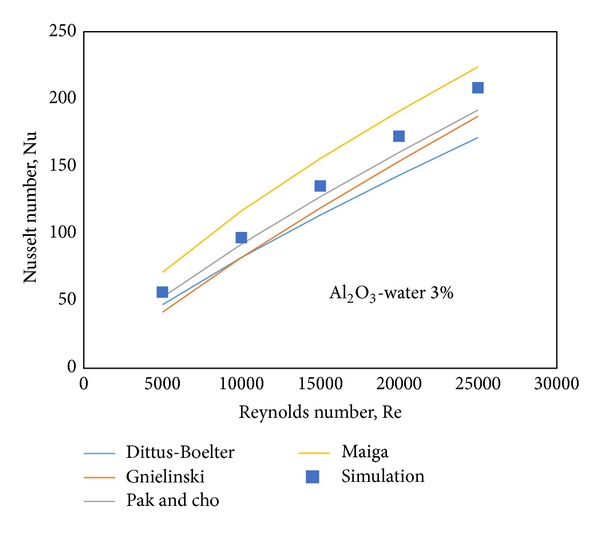
Comparison between the computed data of Nusselt numbers and the data from four benchmarks at different Re numbers for Al_2_O_3_-water 3% nanofluid.

In addition to this, for more comprehensive comparison, the simulation results of Al_2_O_3_-water nanofluid, *ϕ* = 3% and at different Re numbers (5000–25000) are benchmarked against some empirical correlations (Figure 11). Although the simulation results give a good agreement with all the correlations, as expected, the simulation results are between the Pak and Cho correlation and Maiga correlation. The Pak and Cho correlation shows the closest data to the simulation results.

## 4. Conclusions

Numerical simulation of turbulent forced convection heat transfer in a rectangular heated pipe was performed in the present study. The emphasis was given on the heat transfer enhancement resulting from various parameters which include different types of nanofluids (Al_2_O_3_, CuO, SiO_2_, and ZnO), volume fraction of nanoparticles in the range of 1% < *ϕ* < 5% and the Reynolds number in the range of 5000 < *Re* < 25000. The governing equations were solved by utilizing finite volume method with certain assumptions and appropriate boundary conditions to provide a clear understanding of the modeling aims and conditions for the present study. CFD software (ANSYS-FLUENT) has been employed in this study to simulate the current results. The following conclusions could be drawn from the present investigation.

1. Among the investigated nanofluids SiO_2_ generates the highest Nusselt number followed by Al_2_O_3_, ZnO, CuO, and the pure water.
2. Although SiO_2_ has the highest Nusselt number, it has the least heat transfer coefficient because of the lowest thermal conductivity among the tested nanofluids.
3. The Nusselt number increases gradually with the increase of the volume fraction of nanoparticles and Reynolds number.
4. Effect of Reynolds number is more dominant than concentration effect of nanoparticles on heat transfer to nanofluids.
5. The advent of computational fluid dynamic software (Fluent) could provide fair and agreeable result from experimental correlations as noticed in the present research.

## Figures and Tables

**Table 1:** Thermophysical properties of the base fluid and the nanoparticles used in this study [4, 30, 31].

Property	Pure water	Al_2_O_3_	CuO	SiO_2_	ZnO
Density, *ρ* (kg/m³)	998.2	3970	6500	2200	5600
Specific heat, *C_p_* (J/kg*·*K)	4182	765	535.6	703	495.2
Thermal conductivity, k (W/m*·*K)	0.6	40	20	1.2	13
Dynamic viscosity, *μ* (Ns/m²)	0.001003	—	—	—	—

**Table 2:** Comparison between the computed data of Nusselt numbers and the data from two benchmarks at different concentrations for SiO_2_/water.

SiO_2_/water, Re = 15000				
Concentration	Nu (simulation)	Nu (Dittus-Boelter)	Nu (Gnielinski)	Discrepancy between simulation and Gnielinski
1%	134.71	112.83	117.97	12.43%
2%	138.26	116.61	121.76	11.93%
3%	142.64	121.42	126.55	11.28%
4%	149.27	127.47	132.50	11.23%
5%	157.19	135.32	140.13	10.85%

**Table 3:** The comparison between prediction of Nusselt number and benchmarks at *ϕ* = 3% and different Reynolds numbers for SiO_2 _nanoparticles.

SiO_2_/water (3%)				
Re	Nu (Simulation)	Nu (Dittus-Boelter)	Nu (Gnielinski)	Discrepancy between simulation and Gnielinski
5000	59.25	50.41	44.16	25.47%
10000	102.18	87.78	87.31	14.55%
15000	142.64	121.41	126.55	11.28%
20000	181.69	152.83	163.65	9.93%
25000	219.86	182.70	199.32	9.34%

## Nomenclature

### Roman Symbols

*C*_*p*_: Specific heat capacity at constant pressure (J/kg*·*K)
*D*_*h*_: Hydraulic diameter (m)
*d*_*p*_: Nanoparticle diameter (m)
*h*: Heat transfer coefficient based on mean temperature (W/m^2^k)
*I*: Turbulent intensity
*I*_0_: Initial turbulent intensity
*k*: Turbulence kinetic energy (m^2^/s^2^)
k: Thermal conductivity (W/m K)
Nu: Nusselt number (*h·D*_*h*_/*k*)
*p*: Static pressure (N/m^2^)
Pr: Liquid Prandtl number
*q*′′: Heat flux (W/m^2^)
Re_*h*_: Reynolds number (*ρv*_in_ *D*_*h*_/*μ*)
*T*: Temperature (K)
*T*′: Fluctuating part of temperature (K)
*v*: Velocity (m/s)
*v*′: Fluctuating part of velocity (m/s).

### Greek Letters

*ε*: Dissipation rate of turbulence kinetic energy (m^2^/s^3^)
*µ*: Dynamic viscosity (kg/m s)
*µ*_*t*_: Turbulent viscosity (kg/m s)
*ρ*: Density (kg/m^3^)
*ν*: Kinematic viscosity
*ϕ*: Particle volume fraction.

### Subscriptions

eff: Effective
*f*: Fluid
*p*: Particle phase
*s*: Solid
*w*: Wall
*z*: Axial direction
−: Mean
0: Initial.
